# Impact of different fixed flow sampling protocols on flow‐independent exhaled nitric oxide parameter estimates using the Bayesian dynamic two‐compartment model

**DOI:** 10.14814/phy2.14336

**Published:** 2020-01-21

**Authors:** Patrick Muchmore, Shujing Xu, Paul Marjoram, Edward B. Rappaport, Jingying Weng, Noa Molshatzki, Sandrah P. Eckel

**Affiliations:** ^1^ Department of Preventive Medicine University of Southern California Los Angeles CA USA

**Keywords:** Bayesian inference, exhaled breath, FeNO, sampling protocol

## Abstract

Exhaled nitric oxide (FeNO) is an established respiratory biomarker with clinical applications in the diagnosis and management of asthma. Because FeNO depends strongly on the flow (exhalation) rate, early protocols specified that measurements should be taken when subjects exhaled at a fixed rate of 50 ml/s. Subsequently, multiple flow (or “extended”) protocols were introduced which measure FeNO across a range of fixed flow rates, allowing estimation of parameters including C_aw_NO and C_A_NO which partition the physiological sources of NO into proximal airway wall tissue and distal alveolar regions (respectively). A recently developed dynamic model of FeNO uses flow‐concentration data from the entire exhalation maneuver rather than plateau means, permitting estimation of C_aw_NO and C_A_NO from a wide variety of protocols. In this paper, we use a simulation study to compare C_aw_NO and C_A_NO estimation from a variety of fixed flow protocols, including: single maneuvers (30, 50,100, or 300 ml/s) and three established multiple maneuver protocols. We quantify the improved precision with multiple maneuvers and the importance of low flow maneuvers in estimating C_aw_NO. We conclude by applying the dynamic model to FeNO data from 100 participants of the Southern California Children's Health Study, establishing the feasibility of using the dynamic method to reanalyze archived online FeNO data and extract new information on C_aw_NO and C_A_NO in situations where these estimates would have been impossible to obtain using traditional steady‐state two compartment model estimation methods.

## Graphical abstract

A recently developed Bayesian dynamic two‐compartment model of FeNO uses flow‐concentration data from the entire exhalation, which permits estimation of CawNO and CANO from a wide variety of protocols. We compare estimation under single and multiple fixed expiratory flow protocols to demonstrate the gains in precision of multiple flow protocols and the feasibility of estimation with multiple 50 ml/s maneuvers informing, respectively, future study designs and the re‐analysis of archived online FeNO data.

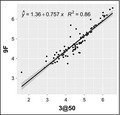

## 1 INTRODUCTION

The fractional concentration of nitric oxide in exhaled breath (FeNO) is an established biomarker used clinically for the phenotyping and management of asthma, with guidelines for assessment (ATS, 1999; ATS/ERS, 2005) and interpretation developed around a standardized exhalation rate of 50 ml/s (FeNO_50_) (Dweik et al., 2012). This is because for any given subject the concentration of NO exhaled depends strongly on the exhalation rate (Hogman et al., 1997; Silkoff et al., 1997). A significant drawback to this approach is that a flow rate of 50 ml/s primarily provides information on NO arising from proximal airway wall sources (George, 2008). Measuring FeNO at multiple flow rates enables estimation of NO sources from distinct anatomical subregions (Eckel et al., 2014; Hogman, Drca, Ehrstedt, & Merilainen, 2000; Pietropaoli et al., 1999; Silkoff, Sylvester, Zamel, & Permutt, 2000; Tsoukias & George, 1998), which may provide added clinical information for the monitoring of rhinitis and asthma (Hogman, 2012; Högman, Malinovschi, Norbäck, & Janson, 2011; Hogman & Merilainen, 2012; Thornadtsson et al., 2015). In 2017, guidelines were updated to include multiple flow FeNO assessment (Horváth et al., 2017). In the commonly employed two‐compartment model (George, Hogman, Permutt, & Silkoff, 2004) the airways are divided into alveolar and airway compartments, and estimates for the contribution from each to FeNO can be calculated.

Multiple flow protocols (George et al., 2004) require observing FeNO at a range of fixed flow rates which is accomplished via 3–10 + exhalation maneuvers, each targeting a different exhalation rate (Molshatski & Eckel, 2017). Often two (or more) replicate exhalations will be performed at each flow rate. In a previous paper (Muchmore, Rappaport, & Eckel, 2017) we have shown that measured flow data can be used to adjust for the effects of flow rate variation, potentially obviating the need for subjects to exercise respiratory control. The dynamic estimation approach introduced in (Muchmore et al., 2017) models FeNO throughout all phases of exhalation. Even when the target is steady exhalation, flow rate variability inevitably occurs during the maneuver since, by definition, the flow rate is 0 when exhalation begins and rises toward the target rate. Participants often overshoot the target, introducing additional variation as they stabilize their flow. Because flow rate variability inevitably occurs during steady‐state protocol measurements, the estimation methods introduced in (Muchmore et al., 2017) can also be used to calculate partitioned estimates of FeNO sources based on a single exhalation, an idea first proposed and demonstrated in (Tsoukias, Shin, Wilson, & George, 2001), or from replicates of maneuvers targeting a single flow rate.

Our work is motivated by existing FeNO data in a large longitudinal study of school children, the Southern California Children's Health Study (CHS). A multiple fixed flow rate FeNO sampling protocol was employed in later years of the study; however, in earlier years only repeated FeNO_50_ data were collected. In this paper, we study how the accuracy and precision of partitioned estimates from the Bayesian dynamic two‐compartment model vary when based upon: a single exhalation targeting a single steady‐state rate, multiple exhalations targeting a single steady‐state rate, and multiple exhalations targeting multiple steady‐state rates. We first explore these issues using simulated FeNO data, for which the true parameter values are known, and we also apply these methods to real FeNO data collected in the CHS. Results from this work will inform the feasibility of extracting significant additional information from archived data by estimating partitioned source estimates from repeated online FeNO_50_ data in the CHS and other studies with archived online FeNO data.

### 1.1 Airway model

#### 1.1.1 Glossary

- *r* and *l* are the airway radius and length (respectively), in cm.
- *z*_0_, *z*_mouth_, and *z*_alv_ are the locations of the sensor, mouth, and alveolar boundary, in cm from *z*_0_.
- *c*(*z*,*t*) is a solution of Equation 1, i.e. the model solution at position *z* and time *t*, in ppb.
- *c_i_*:  = *c* (*z*_0_, *t_i_*) is the model solution at the sensor *z*_0_, ${\hat{c}}_{i}$ is a numerical approximation of *c_i_*, and ${\tilde{c}}_{i}$ is the measured concentration, all in ppb and at time *t_i_*.
- *v* (*t*) is the linear flow rate, in cm/s.
- *d* is the diffusivity of NO in air, in cm^2^/s.
- *p* is the permeability of airway wall tissue to NO, in cm/s.
- *c_w_* is the concentration of NO in the airway wall tissue in ppb.

Using the notation of (George et al., 2004), the parameters we are interested in estimating are C_aw_NO = *c_w_*, the “airway wall concentration”, and C_A_NO = c(*z*_alv_,*t*), the “alveolar concentration” corresponding to the concentration at the alveolar boundary *z*_alv_.

### 1.2 Model description

We employ a dynamic variant of the two‐compartment model (Tsoukias & George, 1998), which is summarized below and was originally described in (Muchmore et al., 2017). The airway is assumed to be a cylinder with fixed dimensions, and there are assumed to be two physiological sources contributing to FeNO. One of these sources is NO producing epithelial tissue lining the airway. The tissue concentration is assumed to be constant throughout the airway and is known as C_aw_NO. As air passes through the airway, NO is assumed to diffuse from tissue to lumen at a rate proportional to the concentration difference.

The dimensions of the alveolar compartment may vary, unlike the airway compartment. However, at any moment it is “perfectly mixed”; that is, the NO concentration is assumed to be constant throughout and is denoted by C_A_NO. While in principle this may vary over time, in practice we assume that it is constant on short (seconds‐minutes) time scales. As described in (Muchmore et al., 2017) the dynamics of this system can be modeled by the partial differential equation (PDE).

(1) $$\frac{\partial }{\partial t}c\left(z,\;\;t\right)=-v\left(t\right)\frac{\partial }{\partial z}c\left(z,\;\;t\right)+d\frac{\partial^{2}}{\partial z^{2}}c\left(z,\;\;t\right)+\frac{2p}{r}\left[c_{w}-c\left(z,\;\;t\right)\right],$$

where C_A_NO is implicitly included as it determines the inflow boundary condition during exhalation. The Bayesian estimation approach we employ necessitates repeated simulation of the underlying physical model, which consists of calculating a series of numerical solutions ${\hat{c}}_{0},{\hat{c}}_{1},\ldots ,{\hat{c}}_{n}$, where exhalation begins at *t*_0_ and ends at *t_n_*. This calculation also requires specification of the velocity function *v*(*t*), which is the only term that varies with time and in a sense “drives” the solution. The fundamental difference between dynamic and steady‐state modeling approaches is that the latter assumes this as constant, while the former (which we employ) uses measured flow data to empirically estimate this function.

## 2 METHODS

### 2.1 Bayesian inference and parameter estimation via Markov Chain Monte Carlo (MCMC)

We consider the problem of parameter estimation in a Bayesian paradigm (Gelman, Carlin, Stern, & Rubin, 2003), and here we outline the approach first described in (Muchmore et al., 2017). Our goal is to characterize the posterior distribution (generically denoted *f*(*θ*|*x*) for parameters *θ* and data *x*) in terms of the likelihood *f*(*x*|*θ*) and a prior distribution *f*(*θ*). In our application the parameters of interest consist of the vector *θ* = (C_aw_NO, C_A_NO), while *x* is the FeNO data collected for each subject. This consists of a collection of measurements denoted $\left\{{\tilde{c}}_{\mathrm{ij}}\right\}$, where ${\tilde{c}}_{\mathrm{ij}}$ is the measured concentration at time *t_i_* during maneuver *j* ϵ 1*,* 2,…. As is often the case, it is sufficient to work with the unnormalized posterior, which simplifies the relationship between the posterior, likelihood, and prior into the proportionality $f\left(\theta |x\right)\propto f\left(x|\theta \right)f\left(\theta \right)$.

We assume that with *θ* fixed the corresponding model equation, and its solution, can be used to calculate the density of the observed data. To formulate a likelihood, we further assume that the observed values ${\tilde{c}}_{\mathrm{ij}}$ arise from a shared parametric conditional distribution with density function *f*, and that conditional on the model solutions *c_ij_* the observed ${\tilde{c}}_{\mathrm{ij}}$ are independent. Then the likelihood can be written as $f\left(x|\theta \right)=\prod_{i}\prod_{j}f\left({\tilde{c}}_{\mathrm{ij}}|c_{\mathrm{ij}}\right)$, where *θ* appears implicitly on the right via the model solution *c_ij_*. Here we assume that ${\tilde{c}}_{\mathrm{ij}}∼N\left(c_{\mathrm{ij}},\sigma \right)$. To efficiently explore the posterior distribution we employ a Metropolis‐Hastings style Markov chain Monte Carlo (MCMC) algorithm (Robert & Casella, 2004), which generically proceeds as follows:

1. Select an initial value *θ* and calculate the likelihood *f*(*θ*|*x*).
2. Propose a new value *θ*′ using a transition kernel $q\left(\theta \to \theta^{\prime }\right)$ and calculate the likelihood *f*(*θ*′|*x*).
3. Accept the proposed value with probability $\min\left[1,\frac{f\left(x|\theta^{\prime }\right)f\left(\theta^{\prime }\right)q\left(\theta^{\prime }\to \theta \right)}{f\left(x|\theta \right)f\left(\theta \right)q\left(\theta \to \theta^{\prime }\right)}\right]$.
4. If the proposal is accepted set *θ* = *θ*′, *f*(*θ*|*x*) = *f*(*θ*′|*x*) then return to 1; otherwise, return to 1.

The efficiency of this type of algorithm can depend crucially on the choice of transition kernel *q*. Although manually finding an optimal *q* may be very difficult, a number of recent MCMC algorithms incorporate an “adaptive” transition distribution (Roberts & Rosenthal, 2009). To account for variability in the posterior across individuals, the adaptive Metropolis algorithm of (Vihola, 2012) is employed to automatically calibrate the proposal distribution, using the implementation provided by (Scheidegger, 2018). This both increases the sampling efficiency while simultaneously automating the choice of transition kernel.

Running the MCMC algorithm described above yields a collection of points {*θ_k_*} known as the posterior sample for the parameter vector *θ*. The point estimates reported herein are maximum a posteriori (MAP) estimators, that is, the estimator that maximizes the posterior probability. We use uniform priors for all parameters, hence in this framework the MAP estimator is equivalent to the maximum likelihood estimator (MLE) on a bounded domain. To quantify uncertainty in the reported MAP estimates the empirical standard deviation of accepted sample points is reported, with the first half of points discarded to account for the “burn‐in” sampling period.

Calculating the MAP/MLE values is an optimization problem, and the MCMC routine can be viewed as a way of approximating the solution. One advantage of the Bayesian approach over deterministic optimization methods is that the posterior sample provides an empirical measure of uncertainty around the estimated values. Probabilistic methods like MCMC may also be less likely than deterministic methods to get stuck at locally optimal solutions, a problem we have encountered when experimenting with deterministic methods for simultaneous estimation of C_aw_NO and C_A_NO.

This modeling framework grants us the flexibility to calculate parameter estimates using either the data from a single exhalation maneuver or data from multiple maneuvers performed by the same subject on the same date. If we assume that each sample from the same individual is independent from the others, then the likelihood of a collection of samples is simply the product of the likelihoods for the individual samples. All MCMC models in this paper run on three chains, each with 5,000 iterations and with different initial values, specifically (100, 1), (200, 2), and (400, 4) for C_aw_NO and C_A_NO respectively. To assess the convergence of the chains we employ the Gelman‐Rubin (Gelman & Rubin, 1992) statistic, denoted by $\hat{R}$, which is a measure of the relative variance within and between chains. Values of $\hat{R}$ near 1 are an indication of convergence, and here we use a threshold of $\hat{R}$ < 1.1 as our convergence criteria. In the simulation study every chain converged during the initial run, although with the real data some samples required additional iterations as discussed in the discussion section.

### 2.2 Southern California children's health study data

Online multiple flow FeNO data were collected at two study visits (2010 and 2011–2012) from more than 1,600 children who participated in the population‐based cohort of the CHS recruited in 2002–2003 from southern California classrooms (McConnell et al., 2006). Characteristics of the cohort have been reported previously (Linn et al., 2013; McConnell et al., 2006). The study protocol (Linn et al., 2009, 2013) requested that participants perform nine constant flow FeNO maneuvers at four target flow rates: three at 50 ml/s, and two each at: 30 ml/s, 100 ml/s, and 300 ml/s. Online samples were collected using ECO MEDICS CLD‐SP Analyzers with DENOX attachment. These devices employ chemiluminescent sensor technology to measure NO concentrations and ultrasound for measuring flow rates.

For this study, we randomly selected 100 CHS participants who performed nine maneuvers at the 2011–2012 visit (as specified in the study protocol) when they were ages 14–16 and had performed at least 2 50 ml/s maneuvers at the previous study visit. To validate use of the Bayesian dynamic two‐compartment model on data arising from various study protocol, we applied it to simulated FeNO data (assuming known C_aw_NO and C_A_NO) based on observed flow data from both study visits and to observed FeNO data from only the 2011–2012 study visit under various study protocol scenarios. The distribution of FeNO_50_ in these 100 participants at the 2011–2012 visit is displayed in Figure S1 (https://doi.org/10.6084/m9.figshare.8968313). The original CHS data collection protocol and this reanalysis of archived data was approved by the University of Southern California Health Sciences campus institutional review board.

### 2.3 Simulated data scenarios

Seven different sampling scenarios were used in the simulation study: four consisted of a single sample at each of the target flow rates employed in the CHS: 30, 50, 100, and 300 ml/s; one scenario consisted of five samples at 50 ml/s (5@50); one scenario consisted of three samples with one each at 30, 100, and 300 ml/s (HMA); and the final scenario consisted of all nine samples specified in the CHS protocol, which includes two samples at 30, 100, and 300 ml/s along with three at 50 ml/s (9F). The single and 9F scenarios were chosen as they represent either the minimal or maximal amount of data available. The HMA scenario was selected as it includes low, medium, and high flow rates, which corresponds to the protocol described in (Hogman et al., 2002) and (Hogman & Merilainen, 2007). Finally, the 5@50 scenario was specified as it corresponds to the sampling scenario employed in earlier years of the CHS. It should be noted that existing approaches require data at multiple flow rates to enable estimation of multiple parameters, thus our ability to estimate multiple parameters based on single flow samples, or multiple samples at single rate, is novel.

To generate a sample of values for the parameters C_aw_NO and C_A_NO to employ in the simulation study, an inverse smoothing spline was created to enable mapping a uniform (0, 1) random variable into random variables representative of current estimates for the corresponding population distributions. These population estimates were taken from the percentiles for individuals under 20 years of age reported in Table 2 of (Hogman et al., 2017), and the (2.5, 5, 25, 50, 75, 95, 97.5) percentiles for C_aw_NO and C_A_NO were (19, 21, 34, 58, 123, 208, 439) and (0.11, 0.61, 1.52, 2.05, 2.73, 3.59, 3.88) respectively. The airway length was assumed to be 25 cm and the airway volume 100 ml, which is downscaled roughly 40% for children based on values of 39.93 cm and 142 ml used in (Condorelli, Shin, Aledia, Silkoff, & George, 2007) for adults. Using these values, the simulated data were generated by solving the model equation numerically via the procedure described in (Muchmore et al., 2017).

### 2.4 Real data scenarios

The scenarios considered when using real FeNO data for estimation were identical to those used in simulations, with one exception. The simulated scenario using five samples at 50 ml/s was chosen as it represents the sampling protocol used in earlier CHS study periods. A larger goal of this work is to enable estimation of both C_A_NO and C_aw_NO using both the 5@50 sampling protocol employed in earlier years along with the 9F protocol employed in later years. This would provide longitudinal estimates of both parameters over a 6‐year period, which when combined with the other data collected during the study could prove very useful for future studies. The possibility of replicating multiple flow estimates using single flow data is considered further in the results section. However, the real data used here employed the 9F protocol, which specifies only three samples at 50 ml/s. Thus, in the real data application the 5@50 scenario is impossible, and instead the closest feasible alternative of 3@50 is adopted.

## 3 RESULTS

### 3.1 Simulated data scenarios

**Figure 1:**
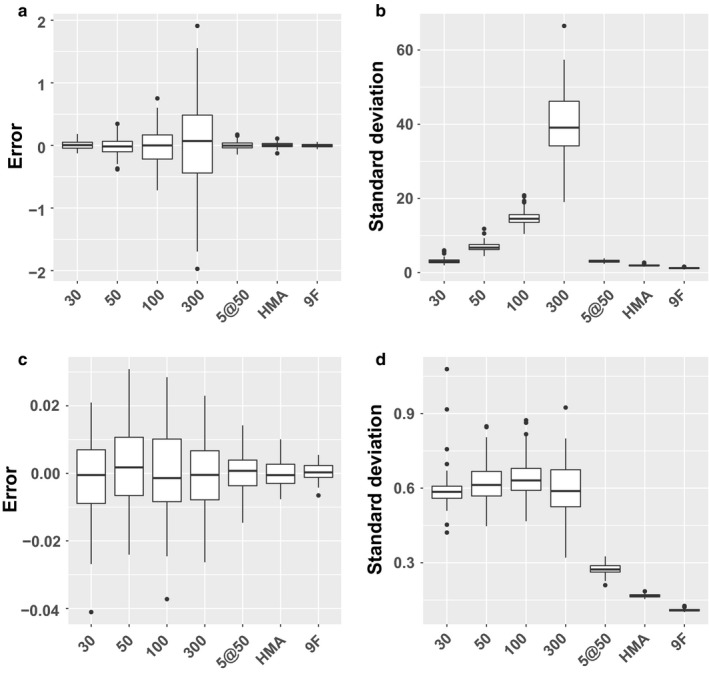
Performance comparison of simulation study results for all scenarios. (a) Distribution of errors as measured by the difference between MAP estimators and values of C_aw_NO used in simulation. (b) Distribution of empirical standard deviation of the MCMC draws after burn‐in estimating C_aw_NO. (c) Distribution of errors for C_A_NO. (d) Distribution of empirical standard deviation for C_A_NO

The accuracy and precision of the estimates for C_aw_NO and C_A_NO with the MCMC estimation procedure applied to the simulated data are shown in Figure 1. Specifically, panels (a) and (c) show the distribution of the estimation error for 100 individuals given each scenario for C_aw_NO and C_A_NO, respectively, while panels (b) and (d) illustrate the standard deviation of the MCMC samples generated during estimation of the respective parameters.

In Figure 1a we see that the error in estimating C_aw_NO appears to be a function of flow rate for the single flow samples, with error variability increasing monotonically as the flow rate increases. The error in estimation arises from the fact that there are many combinations of values for C_aw_NO and C_A_NO which produce model solutions very similar to what is observed. One reason we employ a probabilistic estimation method is because deterministic methods often locate these locally optimal solutions, which can be quite far from the global optima.

For the single flow samples the standard deviation of the posterior samples also increases monotonically as a function of flow rate, as shown in Figure 1b. These empirical standard deviations are a measure of uncertainty in the corresponding parameter estimates, with larger values representing greater uncertainty. Estimates of C_aw_NO based on multiple samples provide more accuracy and precision, and as expected using the full complement of nine maneuvers as in scenario 9F yields the best results. However, differences between the 9F, HMA, and 5@50 scenarios are small. After 9F, HMA provides the next best accuracy and precision, followed by 5@50.

Figure 1c and d illustrate the corresponding results for the C_A_NO parameter. For this parameter there is much less variation in the accuracy and precision of the single flow rate estimates as compared to C_aw_NO. However, there do appear to be differences as the number of maneuvers increases, with small decreases in error variability and larger decreases in posterior sample standard deviation moving from scenarios with a single flow to 5@50, HMA, and 9F.

### 3.2 Real data scenarios

**Figure 2:**
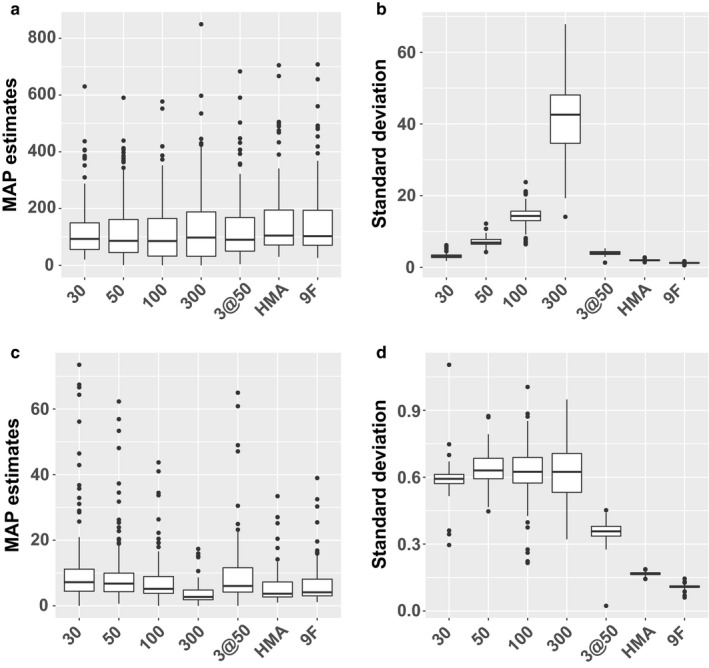
Results from real data application. (a) Distribution of MAP estimates for C_aw_NO. (b) Distribution of empirical standard deviation of the MCMC draws after burn‐in estimating C_aw_NO. (c) Distribution of MAP estimators for C_A_NO. (d) Distribution of empirical standard deviation for C_A_NO

The 100 CHS participants were 57% male, primarily Hispanic (55%) or non‐Hispanic White (31%), 22% reported a doctor's diagnosis of asthma, and had a mean (*SD*) age of: 15.3 (0.6) years, mean (*SD*) height of: 168.5 (8.8) cm, and mean (*SD*) weight of: 63.2 (13.0) kg. Figure 2 illustrates the results when this estimation algorithm is applied to real FeNO samples from the CHS. Because the “true” values for C_aw_NO and C_A_NO are unknown for these samples the estimation error cannot be calculated. Panels (a) and (c) of Figure 2 are plots of the estimates themselves, and unlike Figure 1 there is no a priori reason to prefer one distribution to another. Panels (b) and (d) of Figure 2 are again the standard deviation of the posterior samples, and as in Figure 1, smaller values indicate greater precision.

In Figure 2a we can see that the distribution of C_aw_NO estimates appears to be similar across the various scenarios. To further quantify the degree of agreement between the methods, in the upper portion of Table 1 we provide the Spearman correlation for C_aw_NO estimates from each method. These correlations range from moderate to very strong (*R* = 0.67–0.99), which is consistent with the similarity exhibited by the sample distributions in Figure 2a. However, while the actual estimates may be broadly similar, Figure 2b shows that the sample posterior standard deviations vary considerably. The real data results correspond well with the previous simulated data results. Again there is a monotonic relationship with the estimates based on a single flow rate, and again lower flow rates appear to offer substantially better precision. As before, the multiple flow estimates have more similar precision, again with 9F the best followed by HMA and 3@50.

**Table 1:** Spearman correlation of estimates for C_aw_NO (upper triangular cells) and C_A_NO (shaded lower triangular cells) across all scenarios using real data

Scenario	30 ml/s	50 ml/s	100 ml/s	300 ml/s	3@50	HMA	9F
30 ml/s		0.94	0.87	0.67	0.89	0.94	0.94
50 ml/s	0.81		0.89	0.73	0.94	0.90	0.92
100 ml/s	0.82	0.81		0.76	0.90	0.85	0.86
300 ml/s	0.65	0.70	0.76		0.71	0.67	0.68
3@50	0.80	0.84	0.85	0.75		0.91	0.88
HMA	0.81	0.77	0.81	0.78	0.85		0.99
9F	0.84	0.82	0.84	0.80	0.78	0.99	

Figure 2c illustrates the distribution of C_A_NO parameter estimates, which appear to be somewhat more variable across methods than for C_aw_NO. The correlation between C_A_NO estimates (bottom triangle of Table 1), ranges from 0.65 to 0.99 but the values tend to be lower than the corresponding values for C_aw_NO. As in Figure 1c there is no clear trend in the sample standard deviations for the single flow scenarios. Also similar is the pattern displayed by the multiple flow scenario estimates, with improvement in all three cases and the most marked reduction in *SD* for HMA and 9F.

**Figure 3:**
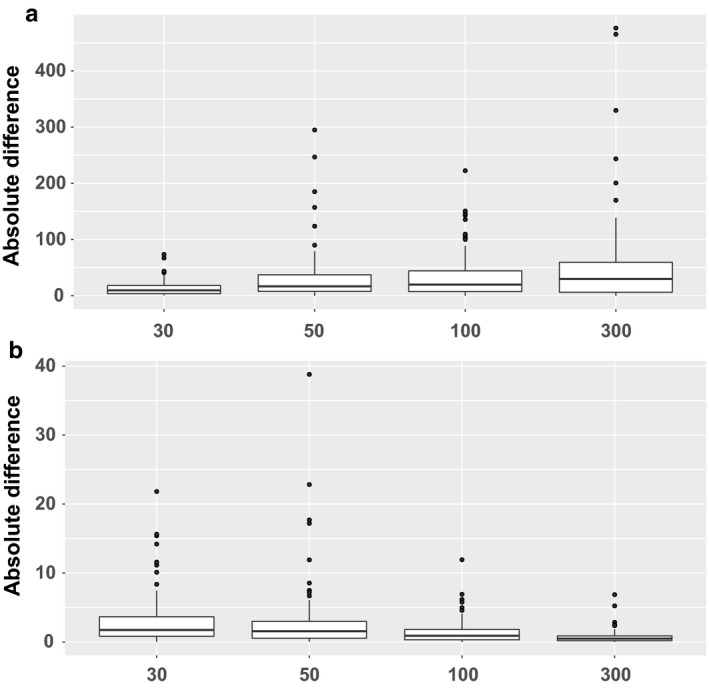
(a) Distribution of absolute differences between MAP estimates of C_aw_NO using two independent samples at the same flow rate. (b) Distribution of absolute differences between MAP estimates of C_A_NO using two independent samples at the same flow rate

The single flow estimates illustrated in Figure 2 were based on a randomly selected maneuver at the appropriate flow rate taken from the nine maneuvers collected during the study. Because replicate samples were collected at each flow rate, another way to assess the quality of the estimates when the true parameter value is unknown is by calculating estimates for each parameter independently for two different samples from the same individual at the same rate. The level of agreement between values indicates the degree of replicability for a given scenario. In Figure 3 the distribution of absolute differences between these two estimates are illustrated at each of the four flow rates, with C_aw_NO shown in Figure 3a and C_A_NO shown in Figure 3b. For C_aw_NO the median difference is smaller at lower flow rates, indicating that estimates based on lower flow rates are more stable. For C_A_NO the pattern is the opposite, with higher flow rates showing more replicability. These single flow results are broadly consistent with the observation that low flow exhalations provide more information regarding proximal sources of NO, while samples collected at high flow rates are more representative of distal NO sources (George, 2008).

To further illustrate the range of estimates within and between individuals, the results for three randomly selected subjects are shown in Table 2. These subjects were selected based on their FeNO_50_ values, representing the 10th, 50th, and 90th percentiles. The single flow estimates are less consistent, reflecting the difficulty in estimating multiple parameters from a single exhalation targeting a steady rate. However, with multiple samples available the consistency in estimation improves, and the HMA and 9F methods estimates are largely similar (recall that in Table 1 Spearman's correlation between HMA and 9F was 0.99 for both C_aw_NO and C_A_NO).

**Table 2:** MAP point estimates and credible intervals across all scenarios for three subjects with FeNO_50_ at the 10th, 50th, and 90th percentiles

Percentile	Scenario	C_aw_NO	95% CI of C_aw_NO	C_A_NO	95% CI of C_A_NO
10	30 ml/s	39.27	(32.79, 46.36)	5.94	(4.57, 7.11)
	50 ml/s	44.78	(27.36, 63.17)	3.35	(1.83, 4.79)
	100 ml/s	20.42	(1.78, 56.77)	3.38	(1.85, 4.24)
	300 ml/s	101.72	(6.44, 161.44)	0.62	(0.05, 1.96)
	3@50	46.73	(40.43, 55.05)	3.73	(2.99, 4.30)
	HMA	55.92	(51.76, 60.44)	1.91	(1.57, 2.24)
	9F	57.31	(54.45, 59.96)	2.22	(2.00, 2.47)
50	30 ml/s	61.65	(56.12, 67.34)	8.90	(7.62, 10.06)
	50 ml/s	39.34	(26.30, 53.28)	9.53	(8.18, 10.73)
	100 ml/s	10.42	(1.08, 48.30)	8.02	(6.31, 8.53)
	300 ml/s	29.82	(2.60, 140.77)	2.68	(0.94, 3.29)
	3@50	48.87	(42.58, 56.97)	8.93	(8.16, 9.54)
	HMA	78.03	(74.19, 81.80)	4.53	(4.20, 4.86)
	9F	79.87	(77.38, 82.25)	4.89	(4.68, 5.14)
90	30 ml/s	309.87	(303.72, 316.44)	12.93	(11.65, 14.05)
	50 ml/s	343.63	(329.44, 356.58)	12.07	(10.81, 13.36)
	100 ml/s	254.04	(223.50, 281.84)	13.50	(12.23, 14.82)
	300 ml/s	206.01	(119.59, 293.91)	7.56	(6.20, 8.87)
	3@50	358.29	(349.07, 365.59)	11.92	(11.22, 12.70)
	HMA	326.61	(322.76, 330.41)	9.30	(8.96, 9.66)
	9F	337.54	(335.83, 340.59)	10.47	(10.20, 10.65)

### 3.3 Relationship between 3@50 and 9F

In the context of the CHS, the relationship between the 3@50 and 9F protocols is of interest. Earlier study years collected multiple FeNO samples at only one rate, while later years collected FeNO at a range of rates; therefore, currently it is only possible to estimate C_aw_NO and C_A_NO for the later years. Here, we have estimated both C_aw_NO and C_A_NO using multiple samples at a single rate (3@50) and a range of rates (9F). We can use these to investigate the relationship between the two scenarios for each parameter and estimate a regression relating the estimates under each scenario.

**Figure 4:**
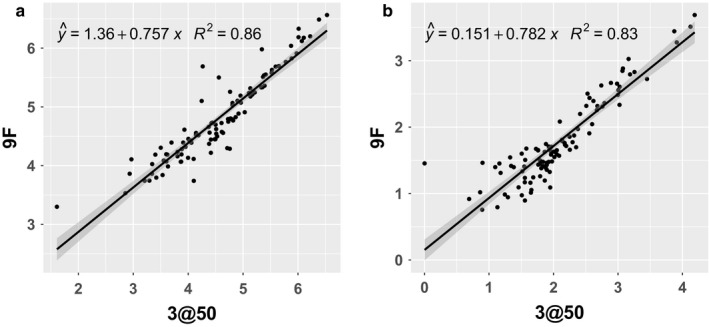
Joint distribution of log1p transformed parameter estimates, and linear regression results relating 3@50 results to 9F estimates for (a) C_aw_NO and (b) C_A_NO

The boxplots in Figure 2a indicate that for all methods estimates of C_aw_NO have a positive skew, and similarly for C_A_NO in Figure 2c, which is not surprising as both are concentrations and therefore must be non‐negative. To understand the joint distribution between estimates, and any potential relationship between them, Bland‐Altman (Bland & Altman, 1986) plots can be found in supplemental Figure S2 (https://doi.org/10.6084/m9.figshare.8198831). To account for the skew, we first log‐transform the data after adding one (log1p), and scatterplots of the resulting values are illustrated in Figure 4. To correct for the lack of agreement between the two methods the best fit linear regression lines and corresponding R^2^ values are also reported in Figure 4, enabling prediction of 9F C_aw_NO and C_A_NO estimates when only repeated samples at 50 ml/s are available.

## 4 DISCUSSION

### 4.1 Summary of findings

In this article, we used simulated data and real multiple flow FeNO measurements from the CHS to evaluate the estimation of C_aw_NO and C_A_NO under a variety of fixed flow rate sampling protocols using the Bayesian dynamic two‐compartment model. We found that both parameters can be reasonably estimated using three or five repeated measures of FeNO at 50 ml/s. As expected, estimation is markedly improved by using multiple flow rates and more modestly improved by including replicates across multiple flow rates. We provide quantification of the improvement, which could inform future fixed flow sampling designs. Our findings suggest that the dynamic model can be applied to repeated online measurements of FeNO_50_, providing new opportunities for re‐analysis of existing archived online FeNO_50_ data.

### 4.2 Comparison with previous literature

The dynamic method applied here is a strict generalization of the steady‐state approach, as proved mathematically in (Muchmore et al., 2017), and the two classes of two‐compartment methods are based on the same underlying physical models and assumptions. However, the dynamic method enables estimation of both C_aw_NO and C_A_NO using data from a single target flow rate (by exploiting information on within‐maneuver variation in flow), a scenario where parameter estimation is impossible for steady‐state methods. Hence the dynamic method parameter estimates reported here are, by design, not meant to be directly comparable with steady‐state results. However, previous results on sampling protocols with steady‐state methods can contextualize our results. In (Molshatski & Eckel, 2017) we investigated the design of steady‐state sampling strategies that are optimal in the sense of minimizing the standard errors of the parameter estimates. For the parameter C_A_NO they conclude that sampling at high flow rates is most important for reducing the associated standard error, while for the parameters D_aw_NO and J’_aw_NO including samples at low flow rates is most effective for reducing the associated standard errors. (Although we do not estimate D_aw_NO and J’_aw_NO directly, C_aw_NO can be expressed as a function of these parameters). Moreover, they find that including some combination of low (below 50 ml/s), medium (between 50 and 200 ml/s), and high (above 200 ml/s) flow rate samples yields optimal results across all criteria for a strategy based on sampling at four different rates. These results are consistent with our results illustrated in Figure 1b and d showing that multiple samples at a range of different rates leads to smaller posterior sample standard deviations than multiple samples at the same rate. They also corroborate our results shown in Figure 3, which illustrates greater consistency of estimates for C_aw_NO at low flow rates, while for C_A_NO estimates at higher flow rates tend to be in closer agreement.

### 4.3 Strengths and limitations

Strengths of this study include a comprehensive statistical evaluation of estimates for C_aw_NO and C_A_NO under various fixed flow sampling protocols with the dynamic model. This includes parallel analyses using simulated and real data, providing both theoretical and practical insight into the statistical properties of the corresponding parameter estimates. We make efficient use of archived online FeNO maneuvers in the CHS, and we take advantage of real flow data from maneuvers in the CHS to generate FeNO profiles in the simulation study. We also establish a relationship between estimates calculated under the 9F and 3@30 protocols, which is of significant interest in the context of the CHS. More generally, we have established that multiple parameters can be estimated from online FeNO data that nominally targets a single flow rate. While this approach is not generally recommended for future data collection, it may provide a way to extract additional information from other existing FeNO studies or may inform the flow rate sampling design for studies of patients with severe airway disease who are unable to perform maneuvers at more extreme (especially low) flow rates (Lázár et al., 2019). Finally, our multiple flow FeNO data were generated using a rigorous data collection protocol on highly accurate ECO MEDICS CLD‐SP analyzers, though other devices simultaneously measuring highly time‐resolved expiratory flow and NO concentration could be used.

This study has several limitations. First, the data collected in the CHS had a digital signal filter automatically applied by the device manufacturer's software (intended to reduce noise in the biofeedback which assists participants in attaining and maintaining target flow rates), so the raw data are not available and cannot be recovered. The signal filtering induces a dependence between consecutive measurements, which is inconsistent with the independence assumption underlying the likelihood. This does not affect the unbiasedness of the point estimates, but it may lead to an underestimate of the associated posterior variance (Gelman et al., 2003). This effect, however, will be counterbalanced by assumptions regarding the error variance discussed below. Second, an underlying assumption of our dynamic model (as in most steady‐state two‐compartment modeling approaches) is that the airway geometry relevant for FeNO dynamics can be represented by a fixed‐size cylinder assumed to be homogenous across individuals. The homogeneity assumption could be relaxed by estimating airway dimensions as part of the MCMC routine, as we are investigating in other ongoing work. A cylindrical airway model also ignores the fact that cross‐sectional volume and surface area increase with airway depth. Previous work with more realistic airway models has shown that a cylindrical model with C_aw_NO which is uniformly distributed significantly understates the effects of back diffusion and underestimates the tissue concentration required in small airways to produce the observed profiles (Shin & George, 2002; Van Muylem, Noel, & Paiva, 1985). Third, in order to estimate C_aw_NO and C_A_NO from single flow rate maneuver(s) we reduced the number of parameters estimated from three as reported in (Muchmore et al., 2017) to only two here. This is accomplished by fixing the airway permeability *p* to the constant value 0.04 derived from the values used in the simulation study (Eckel et al., 2014). Thus, the estimates we calculate are only directly comparable across individuals insofar as this assumption holds true. While it may be reasonable to assume that this coefficient is roughly constant for healthy individuals, diseases like cystic fibrosis likely correspond to significant violations of this assumption. Fourth, in our MCMC estimation routine we fix the standard deviation of the sampling error at *σ* = 5 ppb. However, it is almost certain that this value is much larger than the true sampling error of the instrument, as it is more than two orders of magnitude greater than the zero‐point noise reported in the device specifications. Overestimating the standard deviation results in posterior distributions that are over dispersed or, equivalently, leads to credible intervals that are wider than they would otherwise be (Gelman, 2006). This approach is conservative in the sense that the precision is almost certainly underestimated; however, we chose this value as it also plays an important role in the MCMC routine's sampling efficiency. Specifically, $\hat{R}$ tends to increase as *σ* decreases and vice versa. With *σ* = 5, $\hat{R}$ < 1.1 for all simulated examples and a large majority of real data examples. As a sensitivity analysis we repeated the analysis for the real data examples where $\hat{R}$ > 1.1 with *σ* = 10 and *σ* = 15. In doing so the parameter estimates remain essentially unchanged, and $\hat{R}$ falls below 1.1 in all cases. As another sensitivity analysis, in supplemental Figure S3 (https://doi.org/10.6084/m9.figshare.8198834) we recreate Figure 1 excluding estimates where $\hat{R}$ > 1.1, and they are nearly identical. Finally, we used synthetic versions of the various sampling protocols by considering subsets of archived maneuvers conducted as part of a nine flow protocol. It is conceivable that participants might perform maneuvers differently when independently conducting each sampling protocol, and our analyses do not capture these differences. However, our approach efficiently used archived data.

### 4.4 Conclusions and future directions

In this article, we have demonstrated the feasibility of applying the dynamic method to archived online target flow FeNO data to extract new information on C_aw_NO and C_A_NO in situations where these estimates would have been impossible to obtain using traditional steady‐state two compartment model estimation methods. Given the strong correlation of estimates from the 9F and 3@50 scenarios, these estimates should be useful in population‐level research studies. In future work, we will apply the dynamic method to archived online FeNO_50_ data in the CHS from two study visits. Researchers collecting new online FeNO data for estimating C_aw_NO and C_A_NO, especially for clinical purposes, should collect FeNO at different flow rates. We should also note that the dynamic estimation method is best suited to data from dynamically varying flow exhalation profiles rather than fixed flow rate maneuvers. In future work, we will evaluate the dynamic method applied to data from tidal or other variable flow rate maneuvers. This will require collecting original data from newly recruited subjects, without the various signal processing techniques typically applied by default. A key requirement of new data collection for this purpose is rapid, synchronized sampling of both flow rate and NO concentration; however, limited accuracy is expected and explicitly accounted for in the model description. It has long been recognized that there is potential benefit in optimizing the configuration of existing hardware (Condorelli, Shin, & George, 2004), and there may be potential for continued symbiotic development of sampling and analysis methodologies.

## CONFLICT OF INTEREST

None declared.
