# Supplementary figures and images for: Impact of different fixed flow sampling protocols on flow‐independent exhaled nitric oxide parameter estimates using the Bayesian dynamic two‐compartment model
Source: Physiol Rep. 2020 Jan 21;8(1):e14336. doi: 10.14814/phy2.14336 (PMC6971414; doi:10.14814/phy2.14336)

FeNO50, ppb

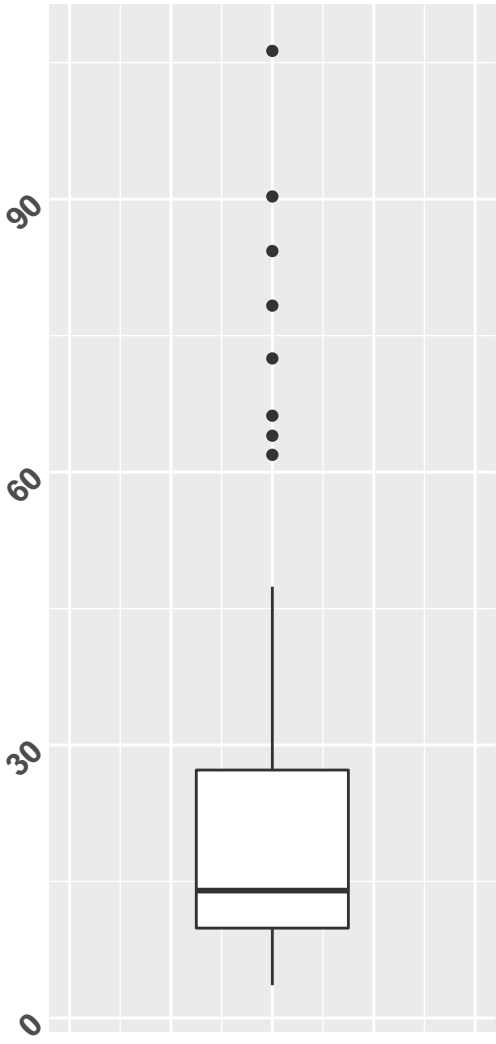

Supplement: Supplementary file 1 [file PHY2-8-e14336-s001.pdf]

**(a)**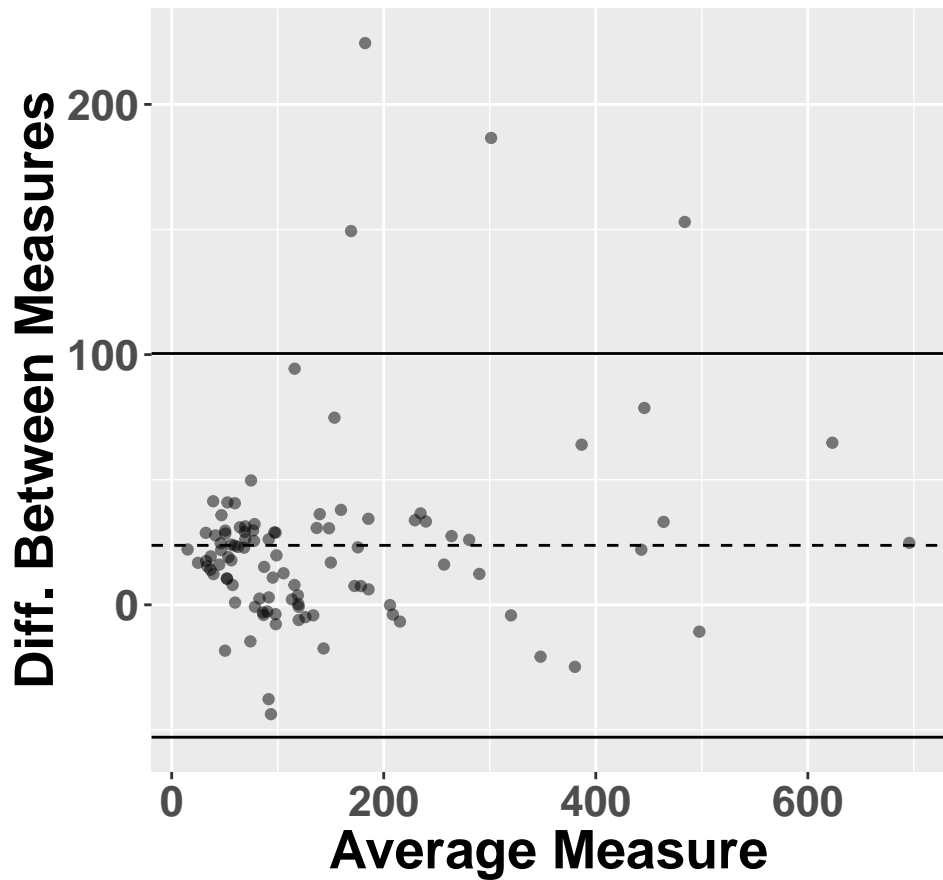**(b)**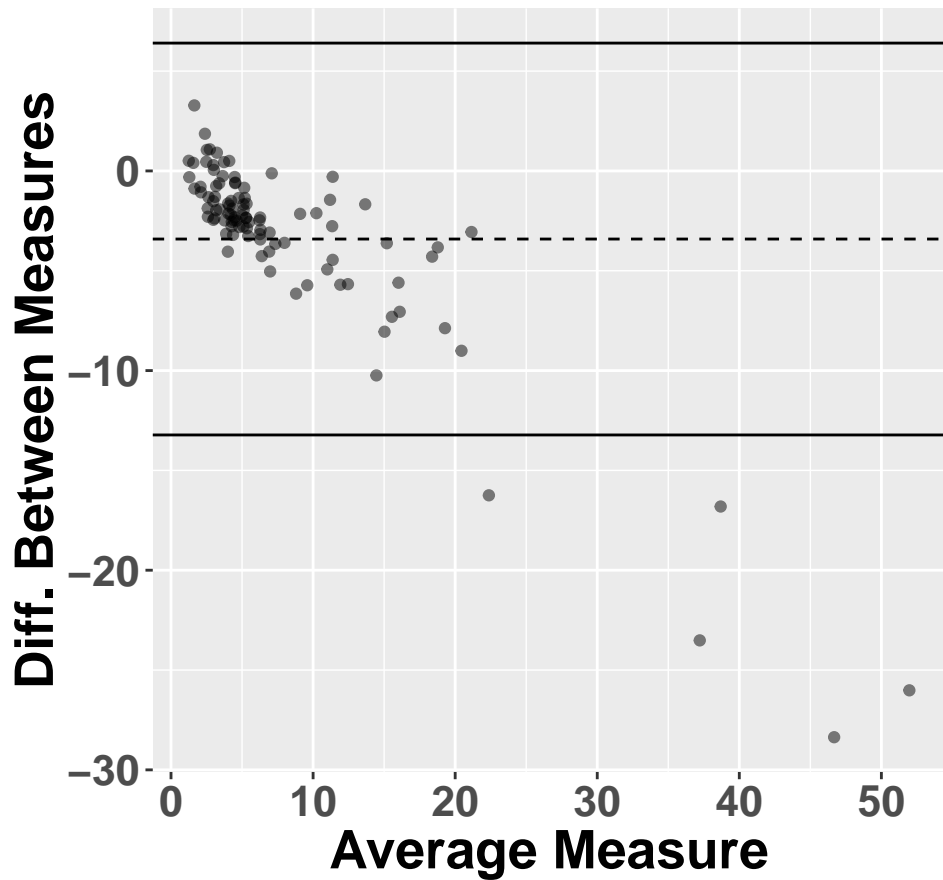

Supplement: Supplementary file 2 [file PHY2-8-e14336-s002.pdf]

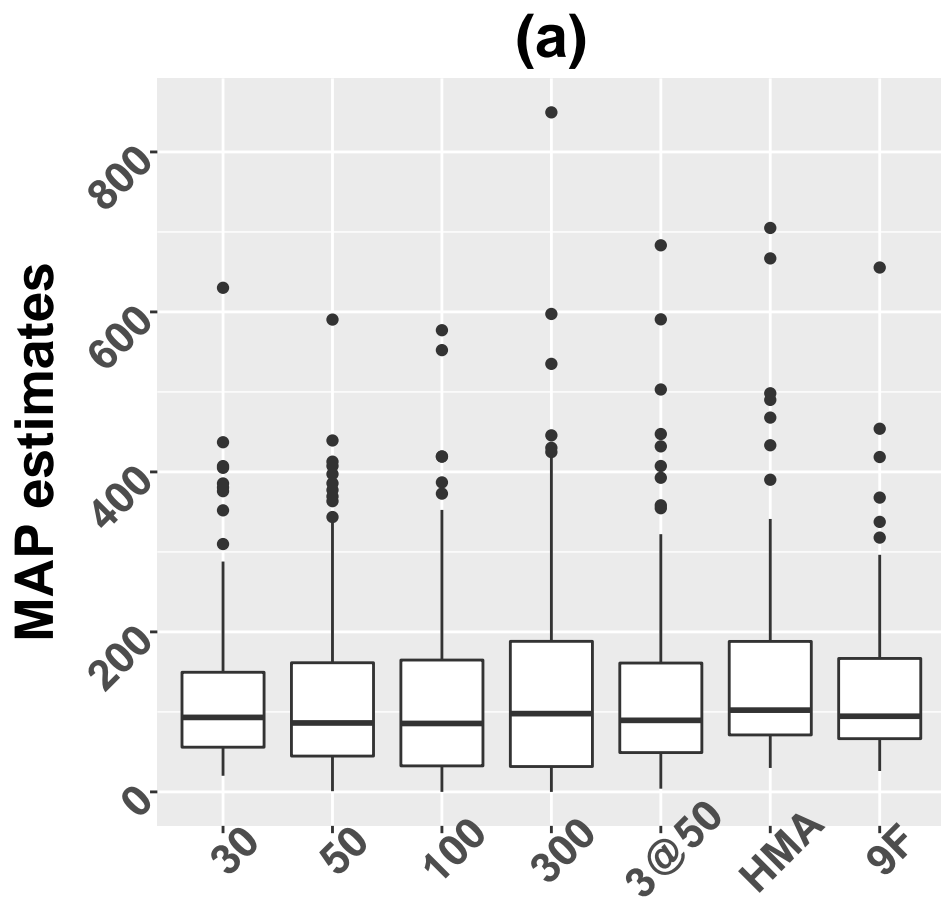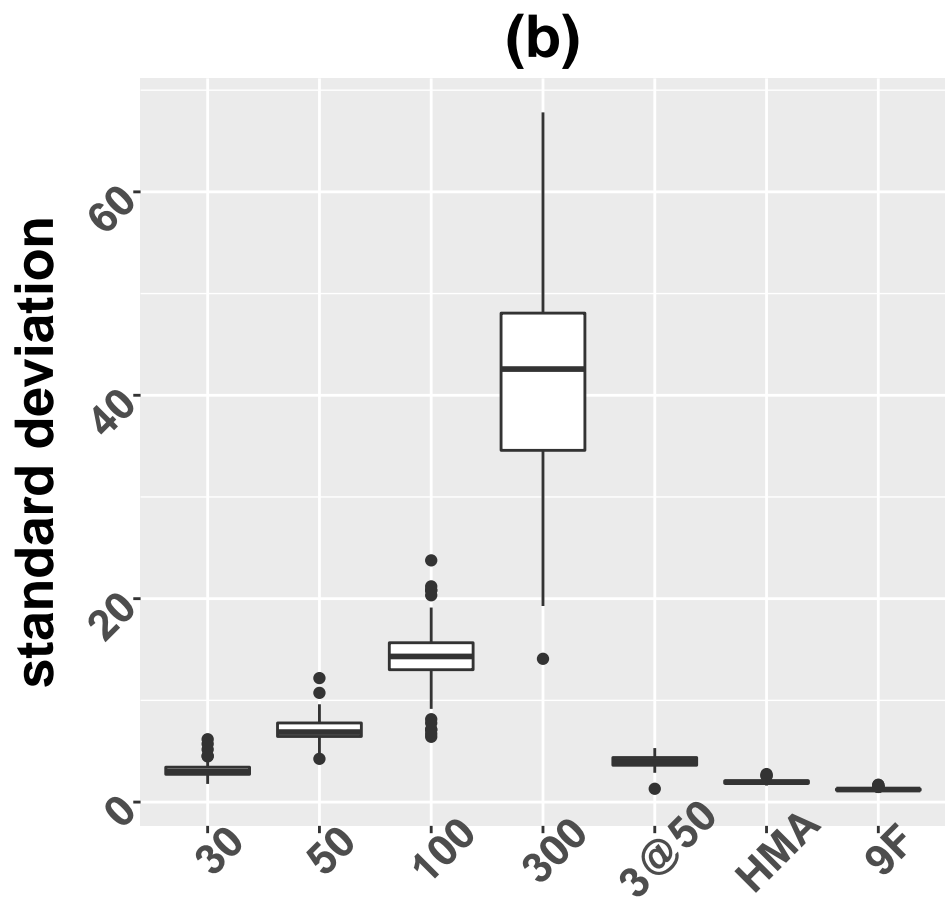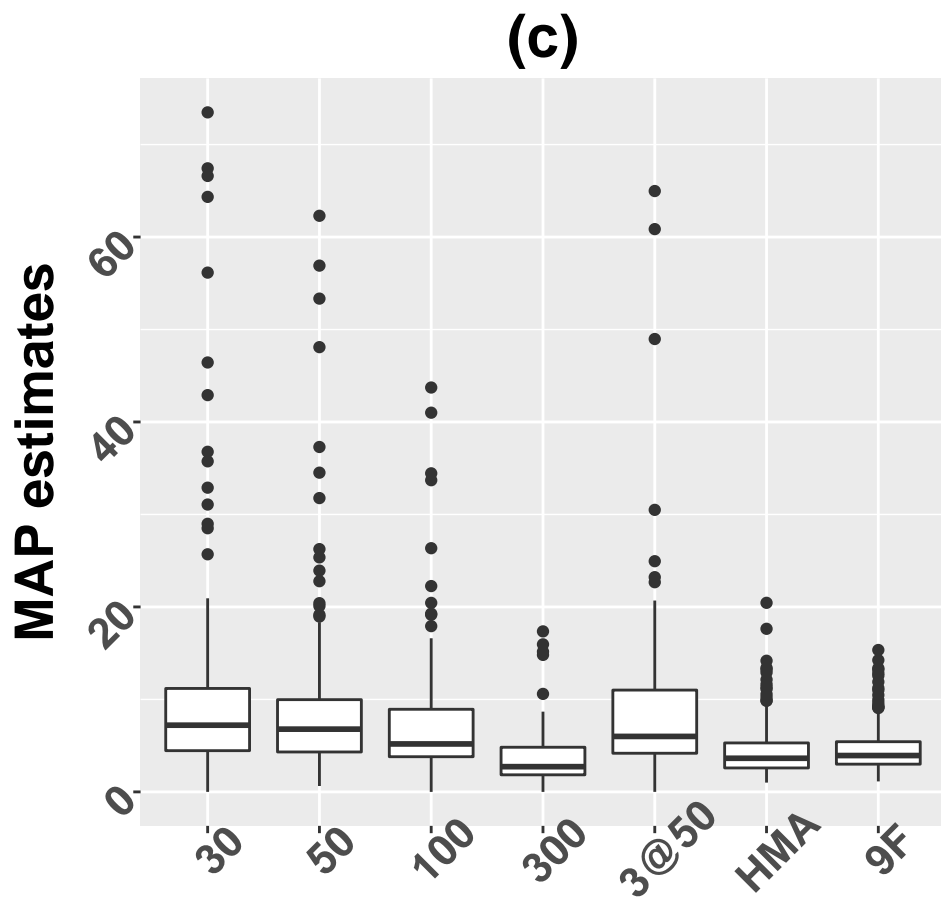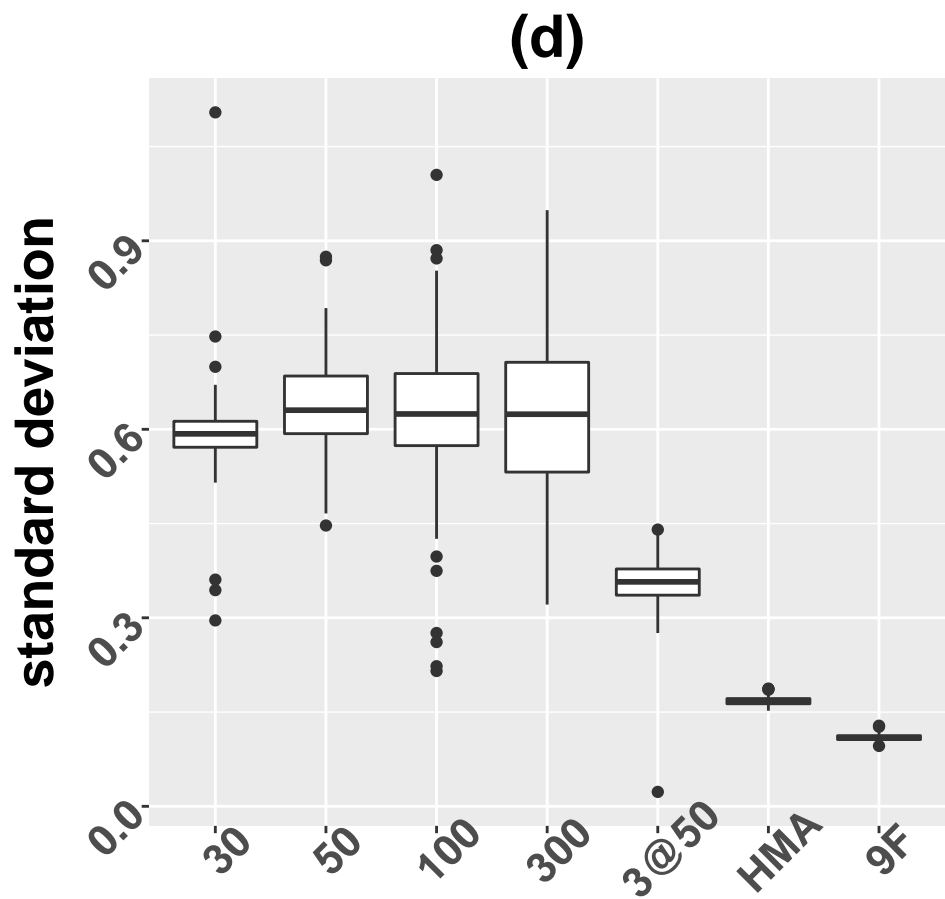

Supplement: Supplementary file 3 [file PHY2-8-e14336-s003.pdf]
